# Evaluation of ensiled soy sauce by-product combined with several additives as an animal feed

**DOI:** 10.14202/vetworld.2020.940-946

**Published:** 2020-05-19

**Authors:** Sadarman Sadarman, Muhammad Ridla, Nahrowi Nahrowi, Roni Ridwan, Anuraga Jayanegara

**Affiliations:** 1 Study Program of Nutrition and Feed Science, Graduate School of IPB University, Bogor, Indonesia; 2 Department of Animal Science, Sultan Syarif Kasim State Islamic University, Pekanbaru, Indonesia; 3 Department of Nutrition and Feed Technology, Faculty of Animal Science, IPB University, Bogor, Indonesia; 4 Research Center for Biotechnology, Indonesian Institute of Sciences, Cibinong, Indonesia

**Keywords:** additive, feed, silage, soy sauce, tannins

## Abstract

**Aim:**

The present experiment aimed to evaluate the use of different additives, i.e., lactic acid bacteria (LAB) inoculant, tannin extract, and propionic acid, on the chemical composition, fermentative characteristics, and *in vitro* ruminal fermentation of soy sauce by-product (SSB) silage.

**Materials and Methods:**

SSB was subjected to seven silage additive treatments: Fresh SSB, ensiled SSB, ensiled SSB+LAB, ensiled SSB+2% acacia tannin, ensiled SSB+2% chestnut tannin, ensiled SSB+0.5% propionic acid, and ensiled SSB+1% acacia tannin+1% chestnut tannin+0.5% propionic acid. Ensiling was performed for 30 days in three replicates, and each replicate was made in duplicate. The samples were evaluated for their chemical composition and silage fermentation characteristics and were tested in an *in vitro* rumen fermentation system.

**Results:**

In general, the nutrient compositions did not differ among the tested SSBs in response to the different additives used. The addition of tannins, either acacia or chestnut, and propionic acid significantly decreased the pH of the ensiled SSB (p<0.05). The addition of several additives (except LAB) decreased the ammonia concentration in SSB silage (p<0.05). The total volatile fatty acids in the *in vitro* rumen fermentation profile of the ensiled SSB were not significantly altered by the various additives applied. The addition of some additives, i.e., ensiled SSB+LAB and ensiled SSB+2% acacia tannin, reduced the digestibility values of the SSB (p<0.05). Different silage additives did not significantly affect methane production, although the addition of acacia tannins tended to result in the lowest methane production among treatments.

**Conclusion:**

The use of additives, particularly 2% acacia tannins, can reduce proteolysis in SSB silage.

## Introduction

There are many underutilized agro-industrial by-products that have the potential for use as animal feeds. One such agro-industrial by-product, soy sauce by-product (SSB), may cause environmental pollution when it is not managed properly [1]. SSB has been reported to contain relatively rich nutrients required by livestock [2]. However, its moisture content is high, which may induce rapid deterioration of the by-product. The conservation of such material is, therefore, necessary to prevent spoilage and to maintain the nutritional quality [3]. Ensiling is a wet preservation technique that is commonly applied in various feed materials. This technique can even improve the digestibility of feed materials by reducing anti-nutritional factors such as trypsin inhibitor [4,5]. Although drying is also a possible technique for preserving SSB, it requires sufficient solar radiation (for sun drying) that might not be achievable during the rainy season or the use of a sophisticated drying facility (for artificial drying).

Soy sauce is characterized by a high-protein content and thus is susceptible to protein degradation and deamination during the silage making process [6,7]. Protein protection in silage is required to maintain protein quality, particularly in high-protein silage such as legume silage and total mixed ration silage. Tannins are a naturally occurring polyphenol that have the ability to interact with other molecules, particularly protein, due to the presence of hydroxyl groups in their structure [8]. A previous study reported that tannin extract from chestnuts could inhibit proteolysis in Moringa and Indigofera leaf silages, as indicated by the lower ammonia (NH_3_) concentration [9]. Furthermore, a meta-analysis revealed that higher tannin levels in the ensiled material resulted in lower NH_3_ and butyrate concentrations [10], indicating its potential application as a silage additive. Other additives that are commonly used for ensuring good quality silage are lactic acid bacteria (LAB) inoculant [11,12] and various organic acids such as propionic acid [13] and formic acid [14,15].

The present experiment aimed to evaluate the use of different additives, i.e., LAB inoculant, tannin extract, and propionic acid, on the chemical composition, fermentative characteristics, and *in vitro* ruminal fermentation of SSB silage.

## Materials and Methods

### Ethical approval

The source of microbial inoculum in this study was rumen fluid that contained solid particles. The rumen fluid was obtained from two fistulated Ongole crossbred bulls before morning feeding time at the Biotechnology Research Center, Indonesian Institute of Sciences, Bogor, Indonesia. The bulls were maintained in accordance with the animal welfare standards of the Indonesian Institute of Sciences. All protocols were approved by the Faculty of Animal Science, Bogor Agricultural University, Indonesia.

### Sample collection and ensiling procedure

This experiment was carried out from September 2019 to February 2020. The SSB was obtained from a company called PT Zebra Bogor, which is located in Cihideung Ilir, Ciampea district, Bogor Regency, West Java Province. A sample of 20 kg (wet basis) was freshly collected and then immediately processed for the ensiling procedure. Acacia bark was obtained from PT Indonesia Fiberboard, Musi Banyuasin Regency, Palembang. The bark was a by-product from the processing of acacia trees for making paper. A total of 50 kg of acacia bark was freshly collected and subsequently transported to the laboratory for further processing. Tannins were extracted from the bark using a hot water extraction system at 120°C, 200 kPa for 3 h. The water was subsequently evaporated in an oven at 50°C for 24 h to obtain tannin powder. The ensiling procedure was performed according to the method of Kondo *et al*. [3] for 30 days. The fresh SSB was weighed, placed on a tray, combined with additives according to the experimental treatments, mixed thoroughly, and stored in a laboratory scale silo. The silo was tightly closed to maintain an anaerobic condition and was placed at room temperature (25-27°C) without any direct sunlight exposure.

### *In vitro* incubation

After being ensiled, the fermented SSB was dried at 50°C for 24 h and ground by a hammer mill to pass through a 1 mm sieve size. All ground samples were incubated *in vitro* with buffered rumen fluid in serum bottles (125 ml capacity) [16]. Each bottle was filled with 500 mg of SSB (dry matter [DM] basis), 17 ml of rumen liquid, and 33 ml of buffer. Before use, freshly collected rumen liquid was filtered with a nylon filter cloth (100 µm sieve size) and then added to the buffer. The rumen buffer solution was saturated with CO_2_ gas for 10 min and subsequently placed into the serum bottle. The incubation bottle was immediately closed and then transferred to a water bath with a temperature of 39°C for 24 h.

### Observed variables

The observed variables included the chemical composition of the fermented SSB (organic matter, ash, crude protein, ether extract, neutral detergent fiber (NDF), and acid detergent fiber [ADF]), the fermentation profiles of the SSB silage (pH, NH_3_, total volatile fatty acid [VFA], and LAB population), and the *in vitro* rumen fermentation characteristics (total VFA, NH_3_, DM digestibility, organic matter digestibility, gas production, and methane). Organic matter, ash, crude protein, and ether extract contents were determined according to the AOAC [17], whereas the NDF and ADF contents were analyzed according to the method of Van Soest *et al*. [18]. These analyses were performed in duplicate from pooled samples, and the data were presented descriptively. The pH of silage was measured using a pH meter, whereas the NH_3_ and total VFA concentrations were determined by Conway microdiffusion and steam distillation methods, respectively [19]. The LAB population was determined by the total plate count method [20]. Gas production was observed 24 h after incubation using a 50 ml gas syringe equipped with a needle. The methane concentration was measured by injecting the gas into a gas chromatograph (Shimadzu 8A GC, Shimadzu Corp., Kyoto, Japan) equipped with a flame ionization detector [21]. To determine digestibility, the samples were further incubated with pepsin-HCl at 39°C for another 24 h [22]. The residue was filtered and dried in an oven at 105°C for 24 h. Digestibility was calculated by subtracting the residue from the initial amount of sample, corrected according to the blank sample.

### Experimental design

The SSB was subjected to seven silage additive treatments: Fresh SSB, ensiled SSB, ensiled SSB+LAB, ensiled SSB+2% acacia tannin, ensiled SSB+2% chestnut tannin, ensiled SSB+0.5% propionic acid, and ensiled SSB+1% acacia tannin+1% chestnut tannin+0.5% propionic acid. The allocation of the treatments into experimental units followed a randomized complete block design with three replications (each was performed in duplicate) for each treatment. Different experimental runs that were performed during different weeks served as the blocks. There were a total of 42 samples (treatment × block/replicate × duplicate).

### Statistical analysis

The data were analyzed with an analysis of variance using IBM SPSS software version 23 (IBM Corp., New York, USA). Duncan’s test was performed for any variables that showed significance at p<0.05 for comparisons among different treatment means.

## Results and Discussion

### Nutrient composition and fermentation characteristics of ensiled SSB

The crude protein content of fresh SSB in the present experiment was slightly above 30% DM (Table-1). This value was comparable to that reported in the study of Uddin *et al*. [23] and higher than that of Yasuda *et al*. [24] who reported CP values of 31.0% and 26.6% DM for SSB, respectively. In general, the ensiling treatments with different additives did not alter the nutrient contents of the SSB. The similarity of the nutrient composition might be caused by the similar raw materials used. In some cases, the difference in raw materials might cause the variations in silage quality. Silage that originates from a raw material with a high-protein content typically has a lower quality than that originating from a raw material with a low-protein content because of its high buffering capacity. For example, it is relatively difficult to ensile legumes and their by-products because of their high-protein contents [25]. In the present experiment, the CP contents of all tested SSBs were comparable, i.e. slightly above 29% DM in response to the different additives applied. The SSB, therefore, showed its potency to be a protein supplement in animal diets. The previous studies have reported the use of Indigofera and Moringa silages as ruminant diets because of their high CP [26,27], and the CP of the SSB in the present study was comparable to those feed ingredients. Ensiling (without any additives) apparently did not change the NDF and ADF contents of SSB. Furthermore, the addition of tannins did not alter the NDF and ADF contents, which has also been reported by other studies [28,29]. Such unchanged NDF and ADF contents in silage indicate the lower affinity of tannins on fiber components compared to protein [9].

**Table-1:** Nutrient composition of ensiled SSB treated with various additives (% DM).

Treatment	Variable					

	OM	Ash	CP	EE	NDF	ADF
R0	74.5	25.5	30.8	15.6	35.5	23.3
R1	78.0	22.0	31.2	16.7	35.6	19.6
R2	79.3	20.7	29.8	16.5	28.0	20.5
R3	77.0	23.0	31.8	14.7	28.8	17.0
R4	77.9	22.1	29.9	18.6	27.6	23.2
R5	77.2	22.8	29.8	17.9	25.3	21.4
R6	79.3	20.7	29.6	23.2	34.2	22.6

OM=Organic matter, CP=Crude protein, EE=Ether extract, NDF=Neutral detergent fiber, ADF=Acid detergent fiber, SSB=Soy sauce by-product, R0=fresh SSB, R1=Ensiled SSB, R2=Ensiled SSB+lactic acid bacteria, R3=Ensiled SSB+2% acacia tannin, R4=Ensiled SSB+2% chestnut tannin; R5=Ensiled SSB+0.5% propionic acid, R6=Ensiled SSB+1% acacia tannin+1% chestnut tannin+0.5% propionic acid

The pH in the ensiled SSB was significantly affected by different additives (Table-2). The highest pH was found in the ensiled SSB without any additives, and it was not significantly different than ensiled SSB+LAB. The addition of tannins, either acacia or chestnut, and propionic acid significantly decreased the pH of the ensiled SSB (p<0.05). The lower pH condition was apparently caused by the increased lactic acid concentration as a result of more water soluble and fermentable carbohydrates. Several bacteria proliferated and used those carbohydrates to generate lactic acid [30]. As more lactic acid is produced, the pH decreases to a greater degree, leading to the growth inhibition of undesired microbes [31]. The addition of appropriate additives to the ensiled SSB is necessary to obtain a higher pH than that of grass [32]. The pH of grass originated silage is approximately 4.0 or lower [29,33], whereas the pH of SSB in the present study was above 5. A previous study stated that it is difficult to obtain a pH of less than 4.5 with legume originated silage [34]. The silage temperature did not differ much among the treatments and ranged from 28.9 to 29.4°C.

**Table-2:** Fermentation characteristics of ensiled SSB treated with various additives.

Treatment	Variable			

	pH	NH_3_ (mmol/l)	Total VFA (mmol/l)	LAB (log cfu/ml)
R1	6.32±0.09^c^	5.13±0.29^c^	46.8±7.65^ab^	6.83±0.23
R2	6.40±0.10^c^	8.67±0.59^d^	68.5±5.79^cd^	6.07±0.42
R3	6.05±0.07^b^	2.61±0.28^a^	38.4±12.61^a^	6.23±0.46
R4	5.88±0.02^a^	2.93±0.44^a^	58.5±7.65^bc^	5.79±0.36
R5	5.90±0.03^a^	5.23±0.49^c^	71.8±2.89^d^	5.83±0.14
R6	5.87±0.03^a^	4.08±0.16^b^	48.4±5.79^ab^	5.91±0.55
p-value	<0.001	<0.001	<0.001	0.070

Means with different superscripts within a column are significantly different (p<0.05). NH_3_=Ammonia, VFA=Volatile fatty acid, LAB=Lactic acid bacteria, SSB=Soy sauce by-product, R1=Ensiled SSB; R2=Ensiled SSB+lactic acid bacteria, R3=Ensiled SSB+2% acacia tannin, R4=Ensiled SSB+2% chestnut tannin; R5=Ensiled SSB+0.5% propionic acid, R6=Ensiled SSB+1% acacia tannin+1% chestnut tannin+0.5% propionic acid

The addition of several additives (except LAB only) decreased the NH_3_ concentration in the SSB silage (p<0.05; Table-2). The lowest NH_3_ concentration was found in the ensiled SSB+2% chestnut tannin, but the concentration did not differ significantly compared with that of ensiled SSB+2% acacia tannin. The decrease in NH_3_ indicated the potency to preserve the SSB protein. During the ensiling process, extensive protein degradation and deamination occur that could lower the protein quality of the feed material [32]. The presence of tannins could protect the protein inside the raw material from microbial degradation. Tannins are composed of free phenolic groups that could generate tannin-protein complexes [35]. Tannins could also impede the activity of proteolytic microbes because they can bind to the specific site of the enzyme that is produced by its bacteria [28]. The reduction of NH_3_ as an effect of the tannin addition was also previously shown by Ding *et al*. [36] during the production of alfalfa silage. Thus, tannins could suppress protein degradation during ensiling, particularly in a high-protein silage such as SSB. The addition of 2% acacia tannins to ensiled SSB was also the only additive treatment that significantly decreased the VFA concentration compared to the original ensiled SSB. This finding was in agreement with a previous study that described the production of Indigofera and Moringa silages [9]. Such lower NH_3_ concentrations in silage are associated with a lower pH, as demonstrated in the present experiment. NH_3_ is considered an alkali and therefore its concentration is positively correlated with silage pH.

### *In vitro* rumen fermentation profiles and digestibility of ensiled SSB

The total VFA in the *in vitro* rumen fermentation profile of the ensiled SSB was not significantly altered by the various additives applied (Table-3). VFA is a fermentation product of carbohydrate [37] that provides more than 70% of the energy supply for the ruminant [38]. Three main VFAs are produced in the rumen: Acetic acid, propionic acid, and butyric acid [38]. The *in vitro* rumen fermentation profile from forages with high-fiber contents could produce high acetic and butyric acid contents, whereas those from concentrated feed material could produce high propionic acid contents [39-41]. A previous study reported that the total VFA depended on the concentration of tannins used and the species of crop that produced the tannins [42,43]. There was no significant effect of several additives on the ensiled SSB in terms of the NH_3_ concentration (Table-3). However, the variations in NH_3_ concentrations observed in this study were within the normal range. The normal NH_3_ concentration in the rumen ranges between 6 and 21 mmol/l [38].

**Table-3:** *In vitro* rumen fermentation profiles of ensiled SSB treated with various additives.

Treatment	Variable			

	Total VFA (mmol/l)	NH_3_ (mmol/l)	IVDMD (%)	IVOMD (%)
R0	109±24.7	16.8±2.74	52.9±1.79^c^	51.3±1.79^c^
R1	121±14.0	15.1±2.16	51.0±1.95^bc^	49.5±1.94^bc^
R2	116±20.7	17.0±4.51	45.3±1.42^a^	43.8±1.43^a^
R3	131±20.9	18.2±1.99	49.1±2.59^b^	47.6±2.59^b^
R4	115±16.0	13.5±2.32	51.2±3.03^bc^	49.6±3.02^bc^
R5	125±27.4	18.0±3.75	52.0±2.09^c^	50.5±2.09^c^
R6	126±15.3	18.1±1.46	53.5±1.22^c^	52.0±1.22^c^
p-value	0.520	0.370	<0.001	<0.001

Means with different superscripts within a column are significantly different (p<0.05). VFA=Volatile fatty acid, NH_3_=Ammonia, IVDMD= *In vitro* dry matter digestibility, IVOMD=In vitro organic matter digestibility, SSB=Soy sauce by-product, R0=Fresh SSB; R1=Ensiled SSB; R2=Ensiled SSB+lactic acid bacteria, R3=Ensiled SSB+2% acacia tannin, R4=Ensiled SSB+2% chestnut tannin, R5=Ensiled SSB+0.5% propionic acid, R6=Ensiled SSB+1% acacia tannin+1% chestnut tannin+0.5% propionic acid

The *in vitro* DM digestibility (IVDMD) and *in vitro* organic matter digestibility (IVOMD) of all tested SSBs were significantly affected by the addition of various additives (Table-3). The addition of some additives reduced the IVDMD and IVOMD values of the SSB, i.e. ensiled SSB+LAB and ensiled SSB+2% acacia tannin (p<0.05). The previous findings reported that the addition of tannins reduced the *in vitro* digestibility of feed materials [44-46]. The reduction of those variables indicated the decrease in nutrient degradation, particularly protein and fiber, since tannins can form complexes with these macronutrients to decelerate their degradation by rumen microbes [8,35]. Such tannin-protein interactions may strategically be used to protect protein from ruminal degradation and enhance the proportion of protein bypass. If the complex is released in the abomasum due to the low pH, protein may then be digested in the small intestine and contribute to the higher metabolizable protein supply for the ruminant livestock [47]. This phenomenon explains the enhancement of milk production and daily gain of dairy cows and beef cattle, respectively, when the animals are administered tannins at an appropriate concentration.

The production of gas and methane in the *in vitro* rumen fermentation of SSB is shown in Table-4. There were no differences in the gas production between fresh SSB and original ensiled SSB (without any additive). However, there was a significant decrease of gas production in the ensiled SSB+2% acacia tannin, the ensiled SSB+0.5% propionic acid, and the ensiled SSB+1% acacia tannin+1% chestnut tannin+0.5% propionic acid compared to fresh SSB (p<0.05). The lowest gas production was observed in the treatment of ensiled SSB+2% acacia tannin. This finding was supported by a previous study that used 1-2% tannin additive on SSB [2]. The gas produced during rumen fermentation is an important variable to evaluate because it could indicate the quantity and proportion of feed consumed by livestock [25,38]. The gas is generated by the process of nutrient degradation in the *in vitro* rumen fermentation system. Carbohydrates contribute the most to gas production, even though protein may also contribute to a lesser extent; fat does not contribute to gas production [48]. Thus, gas production is apparently associated with the carbohydrate contents of the substrate. A previous study showed a positive correlation between starch content and gas production [49].

**Table-4:** The production of gas and methane (CH_4_) in *in vitro* fermented rumen with various additive treatments.

Treatment	Variable	

	Gas production (ml)	CH_4_ (% gas)
R0	26.5±1.72^ab^	2.07±0.40
R1	28.7±0.89^b^	2.24±0.28
R2	28.5±2.48^b^	2.26±0.45
R3	25.3±0.98^a^	1.77±0.45
R4	27.0±0.82^ab^	1.93±0.76
R5	25.8±1.94^a^	1.81±0.77
R6	25.5±1.75^a^	2.22±0.49
p-value	0.010	0.370

Means with different superscripts within a column are significantly different (p<0.05). R0=Fresh SSB, R1=Ensiled SSB, R2=Ensiled SSB+lactic acid bacteria, R3=Ensiled SSB+2% acacia tannin, R4=Ensiled SSB+2% chestnut tannin, R5=Ensiled SSB+0.5% propionic acid, R6=Ensiled SSB+1% acacia tannin+1% chestnut tannin+0.5% propionic acid

The use of different silage additives did not significantly affect methane production, although the use of acacia tannins tended to have the lowest methane production among the treatments (Table-4). Thus, acacia tannins have the potential to impede the growth of methane-forming microorganisms. A previous meta-analysis showed that tannins could reduce the absolute methane content in both *in vitro* and *in vivo* experiments [50]. Two types of tannins are commonly used to reduce methane: Hydrolysable tannins and condensed tannins [38]. Chestnut tannins mainly contain hydrolysable tannins, whereas acacia tannins are primarily comprised condensed tannins. The insignificant methane reduction on ensiled SSB+1% acacia tannin+1% chestnut tannin+0.5% propionic acid is apparently associated with the low concentration of tannins used. The variation of pH values in silages has a minor influence on rumen fermentation parameters such as VFA, NH_3_, and methane in the *in vitro* system. A buffer is typically added in the *in vitro* rumen fermentation system at a certain amount to prevent pH changes so that rumen microbial activity and fermentation can be normally performed until the end of incubation [16].

## Conclusion

The nutrient compositions did not differ among the tested SSBs in response to the different additives used. The addition of acacia tannins or chestnut tannins resulted in decreased pH and NH_3_ concentrations of the SSB silage, thus preserving the SSB during the fermentation process. A reduction of *in vitro* digestibility in the ensiled SSB was observed with the addition of LAB or acacia tannins. The addition of 2% acacia tannins could also reduce the gas production. In conclusion, the use of 2% acacia tannins is recommended for SSB ensiling.

## Authors’ Contributions

SS performed the experiment, collected the data, and drafted the manuscript. AJ designed and supervised the experiment, checked the data analysis, and revised the manuscript. MR, NN, and RR supervised the experiment and revised the manuscript. All authors read and approved the final manuscript.
